# Effect of parental smoking on their children’s urine cotinine level in Korea: A population-based study

**DOI:** 10.1371/journal.pone.0248013

**Published:** 2021-04-15

**Authors:** Myung-Bae Park, Chhabi Lal Ranabhat

**Affiliations:** 1 Department of Gerontology Health and Welfare, Pai Chai University, Daejeon, Republic of Korea; 2 Global Center for Research and Development (GCRD), Kathmandu, Nepal; 3 Manmohan Memorial Institute of Health Science, Kathmandu, Nepal; University of Calfornia San Francisco, UNITED STATES

## Abstract

**Background:**

Children may be exposed to tobacco products in multiple ways if their parents smoke. The risks of exposure to secondhand smoke (SHS) are well known. This study aimed to investigate the association between parental smoking and the children’s cotinine level in relation to restricting home smoking, in Korea.

**Methods:**

Using the Korea National Health and Nutrition Health Examination Survey data from 2014 to 2017, we analyzed urine cotinine data of parents and their non-smoking children (n = 1,403), in whose homes parents prohibited smoking. We performed linear regression analysis by adjusting age, sex, house type, and household income to determine if parent smoking was related to the urine cotinine concentration of their children. In addition, analysis of covariance and Tukey’s post-hoc tests were performed according to parent smoking pattern.

**Finding:**

Children’s urine cotinine concentrations were positively associated with those of their parents. Children of smoking parents had a significantly higher urine cotinine concentration than that in the group where both parents are non-smokers (diff = 0.933, P < .0001); mothers-only smoker group (diff = 0.511, P = 0.042); and fathers-only smoker group (diff = 0.712, P < .0001). In the fathers-only smoker group, the urine cotinine concentration was significantly higher than that in the group where both parents were non-smoker (diff = 0.221, P < .0001), but not significantly different compared to the mothers-only smoker group (diff = - -0.201, P = 0.388). Children living in apartments were more likely to be exposed to smoking substances.

**Conclusion:**

This study showed a correlation between parents’ and children’s urine cotinine concentrations, supporting the occurrence of home smoking exposure due to the parents’ smoking habit in Korea. Although avoiding indoor home smoking can decrease the children’s exposure to tobacco, there is a need to identify other ways of smoking exposure and ensure appropriate monitoring and enforcement of banning smoking in the home.

## Introduction

Smoking causes negative health outcomes not only in smokers but also in people around them, due to the exposure to secondhand smoke (SHS) in all age and sex [1,2]. Efforts to protect non-smokers from SHS exposure were first introduced in the 1970s-1980s [3]. In South Korea, in 1995, the National Health Promotion Act (NHPA) was the first introduction to tobacco control policies. In its early stages, according to the policies, non-smoking areas were separated from smoking areas. In 2006, a new legislation introduced smoking bans in most public and private workplaces, schools, hospitals, outdoor areas surrounding schools, and children’s playgrounds. However, designated smoking areas were still allowed in most other facilities and in most outdoor public spaces. Increased public awareness of smoking risks has led to calls for an expansion of non-smoking areas [4]. Since 2010, the local governments began adding new designated non-smoking areas through local ordinances, such as bus stops, parks, and sidewalks. In 2018, in the most recent national effort, the NHPA was strengthened to include that kindergartens and daycare centers have non-smoking areas within 10 meters from the boundaries of the facilities, and violations of smoking prohibitions carry a fine of up to KRW100,000 (approximately USD80).

Following the Framework Convention on Tobacco Control (FCTC) guidelines by the World Health Organization (WHO), South Korea has had the highest rate of implementation among all tobacco control provisions and one of the highest rates of compliance [5]. Consistent with these public health efforts, the Korea National Health and Nutrition Health Examination Survey (KNHANES) showed that the adult smoking rate in South Korea declined from 25.0% in 2007 to 21.1% in 2017, and the exposure to SHS among non-smokers in the workplace decreased from 46.0% in 2007 to 12.7% in 2017. Even though none of the tobacco control policies specifically focused on private homes, KNHANES also showed a reduction in indoor SHS exposure at home from 14.7% to 4.7% over the same period [6].

The home environment is a particularly important setting for SHS exposure in children. More so, when children live with adults who smoke inside the home or live in multiunit housing where smoking occurs in other units [7,8]. Children may also be exposed to smoking substances as residue in smokers’ homes and buildings, despite indoor smoking ban. Also, their age is transitional due to their physical growth and development [9]. This occurs because tobacco smoke leaves behind a toxic residue (also known as third hand smoke [THS]) on surfaces and in the dust that smokers take into the homes, on their clothes, and on their body. In indoor environments where tobacco is smoked regularly over long periods of time, residual tobacco smoke pollution can become embedded in building materials and may remain on surfaces and in the dust for years, after establishing the smoking ban [10]. There could be some cultural and behavioral factors to SHS. An important issue in South Korea is the association between military service and smoking; since military service is mandatory for all Korean men. Veterans from the South Korean military force describe the military service as a period that strongly influenced them to initiate and maintain smoking [11,12]. Many service members smoke due to the free cigarette distribution and its use at social and cultural functions [13]. This is a social factor that influences the prevalence of tobacco smoking among South Korean men [14]. Even the price of cigarette is low in Korea in comparison to other countries [11]. This situation shows that home and cultural forums would be the high-risk areas to receive SHS for children. Permissive parental attitudes regarding children’s smoking [15], providing weekly pocket money for children by parents [16] would be factors associated with children’s smoking initiation. Considering these situations, home smoking ban may be a misleading indicator for the absence of SHS exposure. Instead, smoking exposure of children should be evaluated based on validated biomarkers that capture the involuntary and often unnoticed exposure to tobacco smoke toxicants from SHS and THS. The majority of previous studies determined the prevalence of smoking by age and sex, and risk factors, using self-administered questionnaires [12], which could be less reliable.

Urine cotinine is a good tool to identify smokers and assess infants and children’s SHS exposure from their parents [17,18]. Although the threshold urine cotinine concentration for identifying smokers is generally below 50 ng/mL [19], in South Korea, the cut-off is even lower (30 ng/mL), and children have a much lower standard at 20 ng/mL [20]. However, children and adolescents tend to have errors in urine cotinine level due to false responses to the question of whether they are smokers [18]. Thus, efforts must be made to avoid measurement errors by excluding false responses.

In this study, the KNAHNES 2014–2018 data on the urine cotinine level in children, measured at the laboratory, were analyzed. The detected urine cotinine could be due to parental smoking. The purpose of this study was to investigate the association between parental smoking and children’s cotinine level in relation to the restricting home smoking, in Korea.

## Materials and methods

### Study design and population

**Fig 1:**
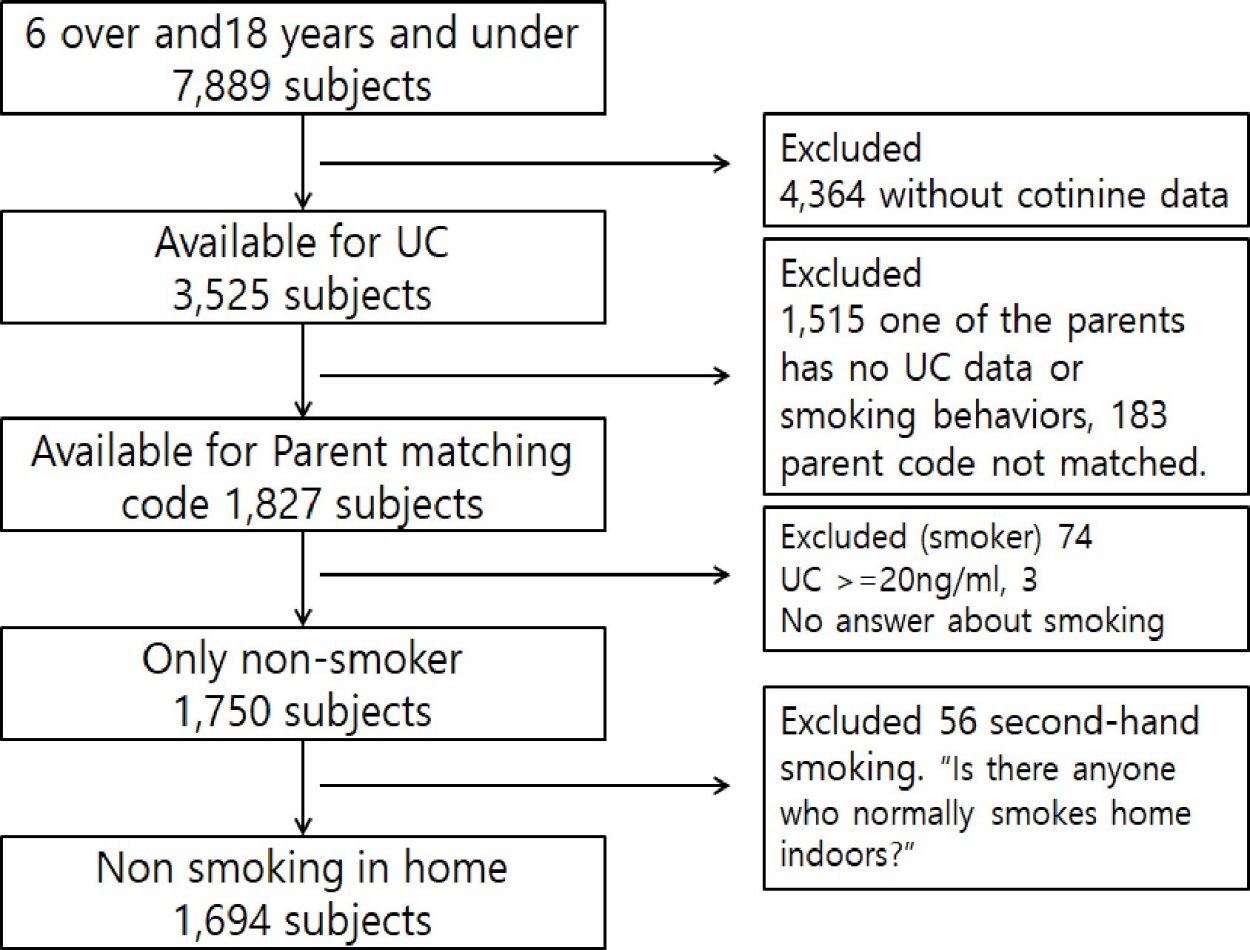
Flow diagram for study subjects.

This study used data from the 2014–2018 KNHANES that yearly assessed about 10,000 people aged ≥1 year. KNHANES utilizes a multi-stratified cluster sampling design where the primary sampling units are the regional units and the secondary sampling units are the households, to select a representative sample from the country. The KNHANES consists of self-reported health questionnaires, nutrition surveys, and physical examinations including blood pressure, pulse, blood test, urine test, and pulmonary function test. This study examined 7,889 non-smoker children aged 6–18 years, of which, 4,364 were without urinary cotinine data, since they did not receive the urine test result. Furthermore, 183 children without a matching parent code; 1,515 with one parent having no urine cotinine data; and those who reported smoking more than one cigarette in the past month (regarded as smokers), were excluded. To avoid measurement errors and prevent the addition of smokers in the analysis, 74 children with urine cotinine concentrations of more than 20 ng/mL that were classified as non-smokers in the raw data were excluded [20]. Finally, to exclude direct smoking exposure at home, the 56 children who answered "yes" to the question "Is there anyone who smoke indoors at home?" were excluded; leaving the final 1,694 children (Fig 1). Furthermore, in Korea, it is highly likely that smokers may under-report smoking behaviors (especially women) [21]; thus, parents with urine cotinine concentrations >50 ng/mL were considered to be smokers.

### Measures

The survey data and biological samples were collected by trained staff in two mobile clinic vehicles. The first vehicle was designed for the physical examinations and the collection of blood and urine samples. The lung function test, eye examination, and the health surveys were administered in the second vehicle. Health surveys included detailed questions about smoking behavior and were conducted by trained interviewers using personal computer (PC) and tablets. The KNHANES subjects were aged from 1 year old; however, with UC, only those aged ≥10 years were investigated in 2015. Since 2016, KNHANES was expanded to include children aged ≥6 years. However, some young children may not have had biochemical data such as urine or blood tests, because these were not examined. Upon collection in the mobile clinic, urine samples were rapidly frozen, transported to a laboratory, and analyzed for cotinine by liquid chromatography–tandem mass spectrometry system on an API 4000 using the TurboIonSpray interface and multiple reaction monitoring (Applied Biosystems/MDS Sciex, Toronto, Canada). For validity and reliability, internal quality controls and external quality program were conducted following the German External Quality Assessment Scheme for Analyses of Biological Materials (G-EQUAS). We reported the creatinine-corrected cotinine levels (nanogram of cotinine per milligram of creatinine); i.e., the cotinine concentration (ng/mL) divided by the creatinine concentration (mg/mL) was used. The limit of detection (LOD) for urine cotinine was 0.2740 ng/mL, while the limit of quantitation (LOQ) was 0.306 ng/mL.

### Data analysis

We performed a descriptive analysis of socio-demographic characteristics and the number of cigarettes smoked per day according to the parents’ smoking patterns. We also calculated the urine cotinine concentrations for the children, mothers, and fathers as geometric means. We performed linear regression analysis by adjusting for age, sex, house type, and household income [7,22] to determine if the parents’ smoking status was related to the urine cotinine concentrations of their children. In addition, analysis of covariance (ANCOVA) and Tukey’s post-hoc tests were performed according to the parents’ smoking pattern at an alpha value of 0.05. All analyses reflected the multi-stratified cluster sampling method, and the correlation and variance analysis included the natural logarithms of the values. All analysis were conducted using SAS for Windows 9.4 (SAS Institute Inc., NC, U.S.A.).

### Ethics approval

The Institutional Review Board of the Korea Center for Disease Control and Prevention reviewed and approved the KNHANES (IRB Nos. 2013-07CON-03-4C, 2013-12EXP-03-5).

## Results

### Participant characteristics

Table 1 describes the socio-demographic characteristics of the children according to the parental smoking pattern. Of the 1,694 children, the parents of 787 (47.1%) were non-smokers. In the homes of 690 (40.8%), 157 (8.9%), and 60 (3.2%) children; fathers only, both parents, and mothers only, respectively, were the only smokers. Mother-only, father-only, and both-parent smokers averaged 4.6 (2.8–6.4) and 14.5 (13.7~15.3), and 7.2 (5.1–9.3) cigarettes per day, respectively.

**Table 1:** Characteristics of parental smoking patterns (number of respondents, percentages).

Respondents (%)		Total	Both non-smoker parents	Mother only smoker	Father only smoker	Both smoker parents
**(N = 1694)**			787(47.1)	60(3.2)	690(40.8)	157(8.9)
**Sex(N = 1694)**	Boy	937(55.2)	423(53.8)	30(49.9)	390(57.2)	94(55.6)
	Girl	757(44.8)	364(46.2)	30(50.1)	390(42.8)	63(44.4)
**Age(N = 1694)**	mean					
	≤12	935(46.3)	414(44.9)	32(43.9)	389(46.5)	100(53.5)
	13–15	418(26.1)	207(27.1)	17(30.7)	159(24.9)	35(24.9)
	16–18	341(27.6)	166(28.0)	11(25.3)	142(28.7)	22(21.5)
						
**House type(N = 1694)**	Apartment	1238(71.8)	606(74.8)	45(71.5)	508(72.2)	79(54.1)
	Other type	456(28.2)	181(25.2)	15(28.5)	182(27.8)	78(45.9)
**House income**	1Q	73(4.1)	36(4.3)	2(3.8)	19(3.0)	16(8.5)
**(N = 1690)**	2Q	375(22.4)	153(20.6)	15(17.9)	155(21.6)	52(36.6)
	3Q	645(38.3)	274(33.8)	32(57.4)	280(43.0)	59(33.8)
	4Q	597(35.2)	322(41.3)	11(20.8)	233(32.3)	30(21.1)
**No. of cig/day of their parent (N = 873)**	Mother	6.4(4.9~7.9)	-	4.6(2.8–6.4)	-	7.2(5.1–9.3)
	Father	15.2(14.5~15.9)	-	-	14.5(13.7~15.3)	18.3(16.7~19.9)

Note: All percentages are weighted.

### Children’s urine cotinine concentrations according to the parents smoking patterns

Table 2 shows the geometric means and 95% confidence intervals (CIs) of the creatinine-adjusted urine cotinine concentration for the entire study population and different subgroups. The concentrations were 9.74 (7.63–12.38), 5.21 (2.94–8.76), 4.06 (3.70–4.45), and 3.04 (2.78–3.31) ng/mg among children whose parents were both smokers, mothers-only, fathers-only, and both non-smokers, respectively. Among the age groups, children aged up to 12, 13–15, and 16–18 years had urine cotinine concentrations of 4.23 (3.85–4.64), 3.78 (3.35–4.25), 3.49 (3.02–4.01) ng/mL, respectively. Regarding house types, the urine cotinine concentrations among those living in apartments and other types of housing were 3.62 (3.30–3.97) and 4.67 (4.12–5.27) ng/mL, respectively. According to household incomes, the urine cotinine concentrations were 5.87 (4.10–8.25), 3.99 (3.40–4.65), 4.07 (3.62–4.55), and 3.48 (3.10–3.89) ng/mL for those in the first to fourth quartiles, respectively. The subgroup values for each variable showed no significant differences within the range of the 95% CI. Among the parent groups, the geometric mean of the mothers’ and fathers’ urine cotinine concentrations were 10.86 (9.36–12.58) and 172.30 (134.89–220.02) ng/mL, respectively. Among the subgroups, the urine cotinine concentration of mothers was 1521.55 (895.75–2584.04), 728.30 (343.02–1545.05), 6.62 (5.95–7.35), and 4.25 (3.87–4.66) ng/mL for both-smoker parents, mother-only smoker, father-only smoker, and both non-smoker parent groups, respectively. The urine cotinine concentration for the fathers groups were 8817.51 (7454.68–10429.47), 4820.32 (4128-72-5627-74), 7.58 (5.79–9.87), and 4.67 (4.27–5.10) ng/mL for both-smoker parents, father-only smoker, mother-only smoker, and both non-smoker parent groups, respectively (Table 2).

**Table 2:** Children’s urine cotinine concentration according to parent’s smoking patterns.

Respondents (%)		Total	Both non-smoker parents	Mother only smoker	Father only smoker	Both smoker parents
**(N = 1694)**	ng/mg (cr)	3.90(3.61–4.20)	3.04(2.78–3.31)	5.21(2.94–8.76)	4.06(3.70–4.45)	9.74(7.63–12.38)
**Sex (N = 1694)**	Boy	3.93(3.61–4.27)	3.12(2.76–3.52)	4.09(2.36–6.71)	3.93(3.55–4.35)	11.23(8.42–14.89)
	Girl	3.85(3.47–4.27)	2.94(2.62–3.28)	6.56(2.96–13.42)	4.24(3.69–4.85)	8.13(5.84–11.19)
**Age(N = 1694)**	-12	4.23(3.85–4.64)	3.03(2.74–3.35)	7.13(2.70–16.88)	4.54(4.05–5.09)	10.52(7.89–13.91)
	13–15	3.78(3.35–4.25)	3.06(2.60–3.59)	3.49(1.34–7.60)	3.79(3.24–4.41)	11.39(7.57–16.89)
	16–18	3.49(3.02–4.01)	3.02(2.50–3.61)	4.75(2.05–9.83)	3.58(2.97–4.28)	6.66(4.10–10.52)
	Apartment	3.62(3.30–3.97)	3.01(2.73–3.53)	5.77(2.81–11.06)	3.82(3.39–4.29)	13.38(9.18–19.31)
**House type(N = 1694)**	Other type	4.67(4.12–5.27)	3.11(2.73–3.53)	3.98(1.80–7.85)	4.73(4.17–5.36)	7.39(5.58–9.70)
	1Q	5.87(4.10–8.25)	4.16(2.96–5.74)	3.55(0.59–12.06)	3.92(2.48–5.96)	26.45(12.31–55.61)
**House income**	2Q	3.99(3.40–4.65)	2.53(2.15–2.97)	5.22(2.21–11.05)	4.52(3.68–5.51)	9.19(5.79–14.29)
**(N = 1690)**	3Q	4.07(3.62–4.55)	3.17(2.74–3.65)	6.06(2.53–13.12)	4.11(3.58–4.70)	10.00(6.91–14.29)
	4Q	3.48(3.10–3.89)	3.09(2.66–3.57)	3.59(1.68–6.85)	3.73(3.25–4.26)	6.79(4.85–9.38)
**UCC of their parents in ng/mL (N = 1694)**	Mother	10.86(9.36–12.58)	4.25(3.87–4.66)	728.30(343.02–1545.05)	6.62(5.95–7.35)	1521.55(895.75–2584.04)
	Father	172.30(134.89–220.02)	4.67(4.27–5.10)	7.58(5.79–9.87)	4820.32(4128-72-5627-74)	8817.51(7454.68–10429.47)

Note: Geometric means and 95% CI.

Creatinine-corrected cotinine.

### Association of urine cotinine concentrations between children, fathers, and mothers

**Fig 2:**
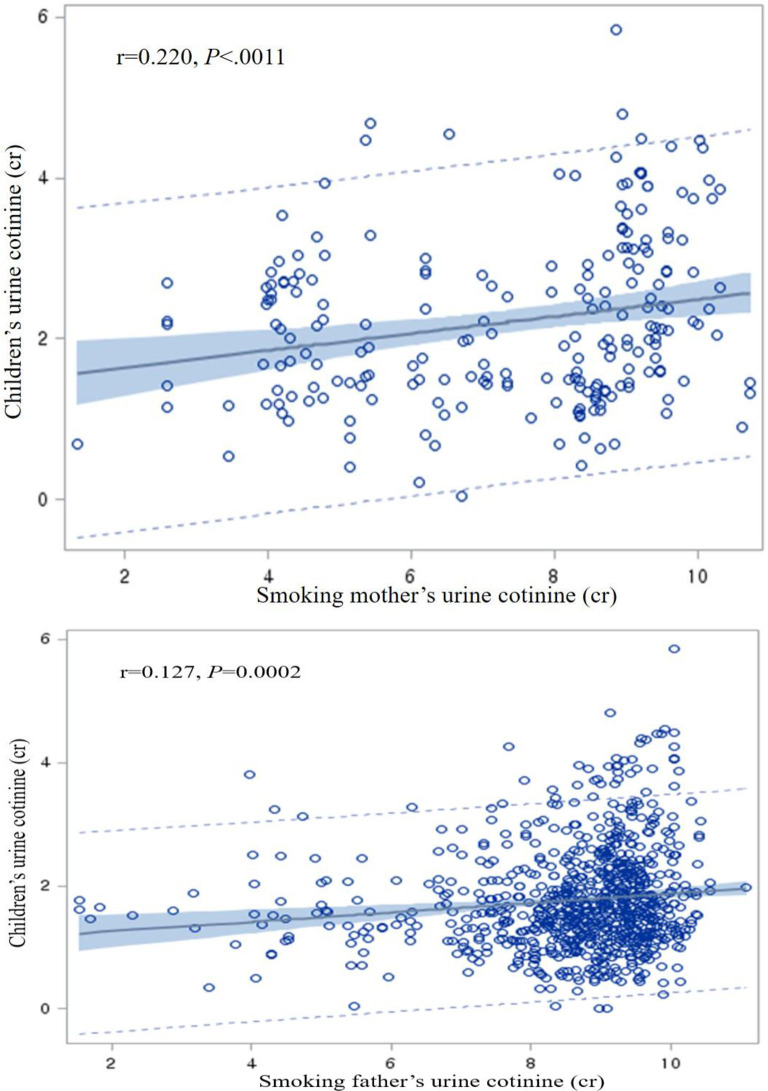
Correlation between log-transformed urine cotinine concentration of children and their parents.

The linear regression analysis was conducted to determine if the independent variables were related to the children’s urine cotinine concentration. In the results of the simple regression analysis, sex and age were not significantly associated with the children’s urine cotinine concentration; but house type, house income, urine cotinine concentration of the father and mother were significantly related (Table 3). We conducted additional correlation analysis to determine the association between smoking parents and the children’s urine cotinine levels. The children’s urine cotinine concentrations were significantly positively correlated with those of their smoking mothers (r = 0.220, *P <* .*0011*) and smoking fathers (r = 0.127, *P* < .0002) (Fig 2). Multiple regression analysis was performed including age, sex, house type, household income, and fathers’ and mothers’ urine cotinine concentrations. The fathers’ and mothers’ urine cotinine concentrations and house type increased positively while age increased negatively with the children’s urine cotinine concentration (Table 3).

**Table 3:** Associations between log-transformed urine cotinine concentrations of children and social-demographic characteristics by linear regression.

	Unadjusted model			Adjusted model		
	B	t	p	B(t)	t	p
Sex*	-0.016	-0.370	0.714	0.004	0.100	0.918
**Children’s Age(continuous)**	-0.015	-2.310	0.021	-0.016	-2.430	0.015
House type*	-0.203	-3.200	0.001	0.121	2.270	0.024
House income*	0.086	2.580	0.010	0.001	0.040	0.969
**UCC of Father**	0.056	8.000	< .0001	0.031	4.600	< .0001
**UCC of Mother**	0.157	9.230	< .0001	0.137	8.070	< .0001
**R-square**				0.186		
**Adj_R-square**				0.183		
**F-value(p-value)**				24.27		< .0001

* References are male, apartment and minimum level income (1Q), respectively.

The urine cotinine concentration was converted a log and analyzed.

Creatinine-corrected cotinine.

**Fig 3:**
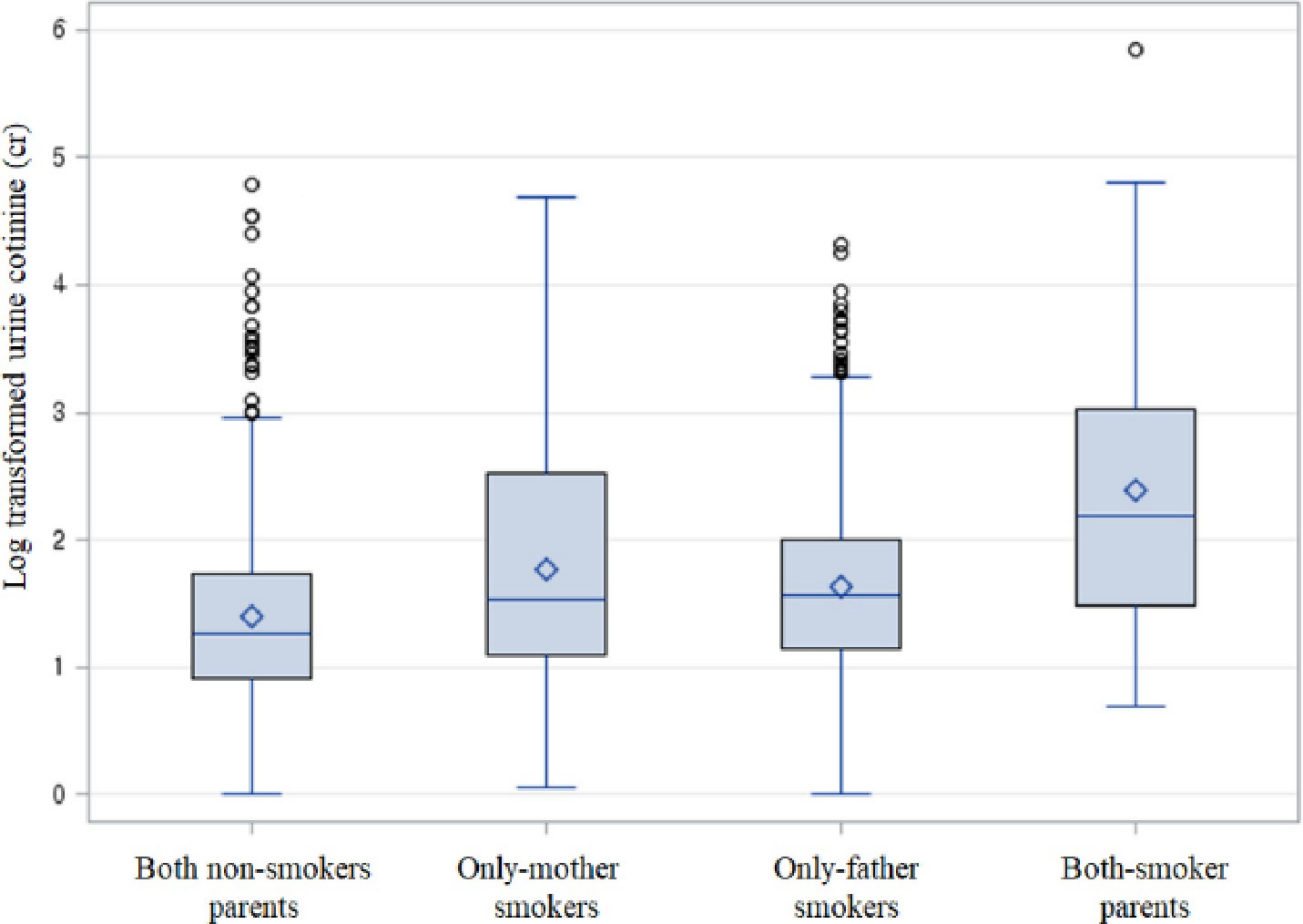
Children’s urine cotinine level by parental smoking status. Both non-smoker parents, mother-only smokers, father-only smokers, and both-parents-smokers by covariance analysis adjusted for age, sex, house type, and household income (alpha = 0.05, *P*<0.0001).

ANCOVA was performed using log-transformed data [23], adjusted for age, sex, house type, and household income. The children’s urine cotinine concentration among the four groups showed statistically significant differences (alpha = 0.05, *P*<0.0001) (Fig 3). Tukey’s post-hoc tests were used to identify statistically significant differences between groups. The group with both parents being-smokers had a significantly higher urine cotinine concentration than those with either both parents being non-smokers (diff = 0.933, *P* < .0001), mothers only being smokers (diff = 0.511, *P* = 0.042), or fathers only being smokers (diff = 0.712, *P* < .0001). In the father-only smoker group, the urine cotinine concentration was significantly higher than that in both non-smoker parent group (diff = 0.221, *P* < .0001); but not significantly different compared to the mother only smoker group (diff = - -0.201, *P* = 0.388). Finally, the mothers’ only smoker group’s urine cotinine concentrations were not significantly different from the non-smoker parents urine cotinine concentration (diff = 0.421, *P* = 0.070) (Table 4).

**Table 4:** Difference in urine cotinine levels according to ANCOVA and Tukey’s post-hoc tests (alpha = 0.05).

Parent smoking status (A)	Parent smoking status (B)	Difference LS means (A)-(B)	P-value	95% Confidence Limits
**Both smoker (3)**	**Both non-smoker (0)**	0.933	< .0001	(0.713–1.152)
**Both smoker (3)**	**Mother only smoker (1)**	0.511	0.042	(0.018–1.004)
**Both smoker (3)**	**Father only smoker (2)**	0.712	< .0001	(0.490–0.934)
**Father only smoker (2)**	**Both non-smoker (0)**	0.221	< .0001	(0.134–0.308)
**Father only smoker (2)**	**Mother only smoker (1)**	-0.201	0.388	(-0.656–0.255)
**Mother only smoker (1)**	**Both non-smoker (0)**	0.421	0.070	(-0.034–0.876)

Note: Adjusted for age, sex, house type, and household income.

Log-transformed.

Creatinine-corrected cotinine.

## Discussion

In this population-based study, we measured urine cotinine levels to confirm the level of exposure to smoking in Korea. The current study assessed the effects of tobacco product exposure in children living in homes with smoking restrictions. This study showed a correlation between parents’ and children’s urine cotinine concentrations; supporting the evidence of home smoking exposure due to the parents smoking status and the likelihood of the children’s exposure to THS.

Previous studies have shown that restricting home smoking reduces the urine cotinine levels [24,25], and this study also supports that finding. Restricting of home smoking is one of the popular tobacco control policies against children and youths’ exposure to tobacco [26]. However, in line with previous findings, the present study also verified that children in Korea living with smokers are exposed to tobacco products [27,28]. Even if the parents smoke outdoors, the smoke contaminants may remain on the parents’ clothes or skin and may be brought home; potentially exposing the children through THS pathways [27,29,30]. In other words, even if smoking is restricted at home, children are likely to be still exposed continuously to smoking substances in the home. Schools are non-smoking areas not only indoors but also outdoors such as playgrounds. Furthermore, in 2018, laws were strengthened to prohibit smoking outside of kindergartens and daycare centers as well as within 10 meters of the premises. Therefore, our research team estimates that these results have more impact at home than anywhere else.

The present study demonstrates higher levels of exposure when both parents smoked than when only one parent smoked. Despite the differences in the targeted age groups, the urine cotinine concentrations in children with smoking parents in this study was lower than that reported for those of the USA infants’ households, contaminated with tobacco smoke; however, similar urine cotinine concentrations were found in children with non-smoking parents and those in the unexposed groups [24]. A study in the UK showed that the highest saliva cotinine level was found in children whose parents smoked most days. Even in smoke-free homes, when parents smoke, the children’s saliva cotinine was found to be higher than that of children of non-smoking parents, similar to a previous report by Jarvis and Feyerabend [31]. In general, the quantity and frequency of cigarette smoked are higher among men than among women [32–34]. Similarly, the number of cigarettes smoked per day by fathers in the present study were nearly 2.6 times higher than those by mothers. The urine cotinine concentration of fathers who smoked was higher than that of mothers who smoked. There was a significantly lower urine cotinine concentration in both non-smoker parent group than in the fathers-only smoker group. Conversely, there was no significant difference between the non-smoking parents’ and mothers-only smoker groups. However, this does not imply that the mothers’ smoking status is irrelevant with respect to the children’s smoking exposure. Despite the high amount of cigarette smoking, the children’s urine cotinine concentration in the fathers-only smoker group was lower than that in the mothers-only smoker group. In general, mothers spend more time looking after their children and doing household activities compared to the fathers. Women also bear heavier responsibilities in caring for the children and in housekeeping, in Asian countries, where Confucianism is widespread, as is the case in South Korea [35,36]. For this reason, mothers are likely to stay at home and have increased contact with their children, with more effects due to smoking exposure at home. Although no statistically significant difference was observed in comparison with both non-smoker parent group, the children of the mother-only smoker group showed a higher urine cotinine concentration than those of the father-only smoker group. This is because the mothers’ urine cotinine concentration had a wider 95% CI than that of the fathers, probably for the following two reasons. First, the number of mothers-only smokers was relatively small; second, the amount of smoking by women varied more than those by men [37]. In other words, women had a greater deviation for the number of cigarettes smoked per day. In this study, the results of the linear regression analysis suggested that the urine cotinine concentration of the children was higher in relation to that of the mothers than that of the fathers. Thus, mothers’ smoking affected their children more. The urine cotinine concentration was highest in the group in which both parents smoked probably because both the mothers and fathers exposed their children to smoking. The urine cotinine concentration of children in both-parent smokers group was higher than that of the mother-only and father-only smoker groups. This finding indicates that the frequency of exposure is likely to be higher when there are two rather than one contamination sources of smoking exposure. In addition, the amount of smoking in the present study was also higher in both-parent smoker group, where the fathers and mothers smoked an average of 19.5 and 6.2 cigarettes, respectively. The father-only smoker group smoked 14.4 cigarettes on the average, while the mother-only smoker group smoked 5.2 cigarettes. Thus, children whose parents both smoked were more likely to have more frequent exposure to higher concentrations of SHS or THS.

Most parents, even those that smoke, do not want their children to be exposed to passive smoking because they know that it is harmful to their children [38]. For this reason, most parents avoid smoking indoors to prevent their children from being exposed to passive smoking [29]. However, children may be influenced by the smoking of their parents and seniors from social functions and peer group gathering in picnic, hiking, trekking etc., in Korea. One study verified that even if the smoking parents made efforts to prevent passive smoking, their house and infants were still exposed to THS [27]. The present study also demonstrated that even if the parents avoided smoking indoors, their children were exposed to higher concentrations of smoking substances. In Korea, children spend most of their days at school and home, and facilities for children and youths are subject to strict regulations on smoking. For this reason, the level of exposure to SHS is assumed to be relatively low in these public places. The present study found that children are exposed to smoking substances even when their smoking parents tried to prevent passive smoking. Children are more vulnerable to smoking exposure at home since they breathe quickly, spend more time at home, and have relatively thin skin [39]. Although avoiding smoking at home can decrease the children’s exposure to smoking [27,40], it is impossible to completely prevent the exposure. There is no safe level of exposure to tobacco smoke [41]. Parents must abstain from smoking in order to prevent passive smoking by their children [42]. Parents’ smoking itself is a threat to their children’s health.

Finally, in our study, interestingly, the concentration of urine cotinine in the apartments was higher, and this is the variable that was related to the children’s urine cotinine concentration after that of the mothers. This is in agreement with the results of previous studies, showing that more exposure to secondary smoke occurs in multi-unit housings [31,43]. In addition, due to the characteristics of apartments that are physically closer to others and apartments with toilet vents, it may not be possible to identify smoke from other houses as the source of SHS. Majority of Korean people including the young prefer to live in apartment. Thus, the house type is an important variable for this study.

### Policy implications

More than any other population, children must be strictly protected from smoking. Even though smoking is banned at the homes to protect children from SHS; however, it is still highly likely that children are exposed to smoking at home by THS. The best way to prevent THS at home is to educate, if a household includes a smoker, regarding THS and on how to abstain from smoking [24]. The home should be the optimal place for children to be protected from harm. Parents must understand that their smoking inevitably exposes their children to carcinogens [44,45]. Lastly, even if the parents quit smoking, cleaning of the house with no smoking can help reduce the exposure to THS at home because tobacco chemicals can exist in the house for a long time [46].

### Limitations of study

This study clearly described the association between parents smoking and children’s urine cotinine but there are some limitations. This study did not completely exclude the exposure to SHS beyond the home. In the KNHANES, children were asked about their SHS exposure in public places other than their homes. Excluding SHS exposures in public places as well as the non-responses to questions did not change the results of this study (See S1–S3 Tables). Since children could not be forced to provide responses to this question to avoid making them vulnerable, some participants did not respond to it. Thus, it was not made a required response. Because there were many non-responses, excluding them could cause response bias. In addition, only cigarette smoking was examined in this study. The findings from the study should be carefully generalized and further studies are recommended considering those limitations.

### Conclusions

The results of this study verified that the parents’ smoking is a crucial factor for children’s urine cotinine and there is need to carefully update household smoking ban policy. This study showed a correlation between parents’ and children’s urine cotinine concentrations, supporting the occurrence of home smoking exposure due to the parents’ smoking habit in Korea. Although avoiding home indoor smoking can decrease the children’s exposure to smoking, parents must reduce the amount of smoking, or better still to quit smoking.

## Supporting information

S1 Table Characteristics of parental smoking patterns. (DOCX)

S2 Table Children’s urine cotinine concentration according to parent’s smoking patterns (Unit: ng/mg). (DOCX)

S3 Table Difference in urine cotinine levels according to ANCOVA and Tukey’s post-hoc tests (alpha = 0.05). (DOCX)

S1 Data (CSV)

S2 Data (ZIP)
