# Correction: Effect of parental smoking on their children’s urine cotinine level in Korea: A population-based study

**DOI:** 10.1371/journal.pone.0256412

**Published:** 2021-08-16

**Authors:** Myung-Bae Park, Chhabi Lal Ranabhat

The following information is missing from the Funding statement: This work was supported by the National Research Foundation of Korea (NRF); the Korea government (MSIT) (No. NRF-2020R1C1C1007913).
