# Supplementary material for: Effect of parental smoking on their children’s urine cotinine level in Korea: A population-based study
Source: PLoS One. 2021 Apr 15;16(4):e0248013. doi: 10.1371/journal.pone.0248013 (PMC8049314; doi:10.1371/journal.pone.0248013)
Supplement: S1 Table — (DOCX) [file pone.0248013.s001.docx]

**STable 1. Characteristics of parental smoking patterns**

| **Respondents (%)** |  | **Total** | **Both non-smoker parents** | **Mother only smoker** | **Father only smoker** | **Both smoker parents** |
| --- | --- | --- | --- | --- | --- | --- |
| **(N=1010)** |  |  | 466(47.0) | 29(2.5) | 430(43.2) | 85(7.4) |
| **Sex (N=1010)** | Boy | 546(53.8) | 239(44.7) | 13(2.0) | 239(44.9) | 55(8.4) |
|  | Girl | 464(46.2) | 227(49.7) | 16(3.0) | 191(41.2) | 30(6.1) |
| **No. of cig/day of their parent (N=379)** | Mother | 6.8(4.1-9.5) | - | 5.1(2.4-7.8) | - | 7.7(4.4-11.0) |
|  | Father | 14.9(14.1-15.8) | - | - | 14.3(13.4~15.2) | 18.7(16.7~20.7) |

Note: All percentages are weighted.
