# Supplementary material for: Effect of parental smoking on their children’s urine cotinine level in Korea: A population-based study
Source: PLoS One. 2021 Apr 15;16(4):e0248013. doi: 10.1371/journal.pone.0248013 (PMC8049314; doi:10.1371/journal.pone.0248013)
Supplement: S2 Table — (DOCX) [file pone.0248013.s002.docx]

**STable 2. Children’s urine cotinine concentration according to parent’s smoking patterns (Unit: ng/mg)**

| **Respondents(%)** |  | **Total** | **Both non-smoker parents** | **Mother only smoker** | **Father only smoker** | **Both smoker parents** |
| --- | --- | --- | --- | --- | --- | --- |
| **(N=1010)** |  | 3.15(2.86-3.47) | 2.37(2.11-2.66) | 3.13(1.95-4.78) | 3.59(3.18-4.04) | 7.75(5.65-10.50) |
| **Sex** | Boy | 3.19(2.85-3.56) | 2.33(1.98-2.73) | 3.25(2.14-4.76) | 3.50(3.04-4.00) | 8.59(5.68-12.76) |
|  | Girl | 3.11(2.76-3.49) | 2.42(2.12-2.74) | 3.04(1.44-5.69) | 3.70(3.12-4.36) | 6.54(4.30-9.74) |
|  |  |  |  |  |  |  |
| **Urine Cotinine Concentration of their parents in ng/mL** | Mother | 8.58(7.09-10.35) | 3.50(3.10-3.93) | 901.45(240.10-3376.98) | 4.98(4.42-5.60) | 4186.27(2593.80-6756.08) |
|  | Father | 169.53(123.94-231.75) | 3.59(3.21-4.01) | 9.18(5.70-14.47) | 5234(4342.25-6309-85) | 8646.84(6835.24-10938.50) |

Note: Geometric means and 95% CI, Creatinine-corrected cotinine
