# Supplementary material for: Effect of parental smoking on their children’s urine cotinine level in Korea: A population-based study
Source: PLoS One. 2021 Apr 15;16(4):e0248013. doi: 10.1371/journal.pone.0248013 (PMC8049314; doi:10.1371/journal.pone.0248013)
Supplement: S3 Table — (DOCX) [file pone.0248013.s003.docx]

**STable 3. Difference in urine cotinine levels according to ANCOVA and Tukey’s post-hoc tests (alpha=0.05)**

| **Parent smoking status**  **(A)** | **Parent smoking status**  **(B)** | **Difference LSmeans**  **(A)-(B)** | **P-value** | **95% Confidence Limits** |
| --- | --- | --- | --- | --- |
| **Both smoker (3)** | **Both non-smoker (0)** | 0.862 | <.0001 | (0.593-1.131) |
| **Both smoker (3)** | **Mother only smoker (1)** | 0.687 | 0.001 | (0.292-1.082) |
| **Both smoker (3)** | **Father only smoker (2)** | 0.551 | <.0001 | (0.270-0.831) |
| **Father only smoker (2)** | **Both non-smoker (0)** | 0.312 | <.0001 | (0.201-0.422) |
| **Father only smoker (2)** | **Mother only smoker (1)** | 0.136 | 0.415 | (-0.192-0.464) |
| **Mother only smoker (1)** | **Both non-smoker (0)** | 0.175 | 0.283 | (-0.146-0.497) |

Note: Adjusted for age, sex, house type, and household income.

Log-transformed

Creatinine-corrected cotinine
